# Effects of tadalafil once daily or on demand versus placebo on time to recovery of erectile function in patients after bilateral nerve-sparing radical prostatectomy

**DOI:** 10.1007/s00345-014-1377-3

**Published:** 2014-08-26

**Authors:** Ignacio Moncada, Fermín R. de Bethencourt, Enrique Lledó-García, Javier Romero-Otero, Carmen Turbi, Hartwig Büttner, Carsten Henneges, Juan I. Martinez Salamanca

**Affiliations:** 1 Department of Urology, Hospital La Zarzuela, Madrid, Spain; 2 Department of Urology, Hospital “La Paz”, Madrid, Spain; 3 Departament of Urology, Hospital General Universitario Gregorio Marañón, Madrid, Spain; 4 Department of Urology, Hospital Universitario 12 Octubre, Madrid, Spain; 5 Eli Lilly and Company, Madrid, Spain; 6 Lilly Deutschland GmbH, Bad Homburg, Germany; 7 Department of Urology, Hospital Universitario Puerta de Hierro-Majadahonda, Madrid, Spain

**Keywords:** Nerve-sparing prostatectomy, Penile function, Phosphodiesterase 5 inhibitors, Prostate cancer, Rehabilitation, Tadalafil once a day

## Abstract

**Purpose:**

We report time to erectile function (EF)-recovery data from a multicenter, randomized, double-blind, double-dummy, placebo-controlled trial evaluating tadalafil started after bilateral nerve-sparing radical prostatectomy (nsRP).

**Methods:**

Patients ≤68 years were randomized post-nsRP 1:1:1 to 9-month double-blind treatment (DBT) with tadalafil 5 mg once daily (OaD), 20 mg tadalafil on demand (“pro-re-nata”; PRN), or placebo, followed by 6-week drug-free washout (DFW) and 3-month open-label OaD treatment. Secondary outcome measures included Kaplan–Meier estimates of time to EF-recovery (IIEF-EF ≥ 22) during DBT (Cox proportional hazard model adjusting for treatment, age, and country).

**Results:**

A total of 423 patients were randomized to tadalafil OaD (*N* = 139), PRN (*N* = 143), and placebo (*N* = 141); 114/122/155 completed DBT. The proportion of patients achieving IIEF-EF ≥22 at some point during DBT with OaD, PRN, and placebo was 29.5, 23.9, and 18.4 %, respectively. DBT was too short to achieve EF-recovery (IIEF-EF ≥ 22) in >50 % of patients; median time to EF-recovery was non-estimable. Time for 25 % of patients to achieve EF-recovery (95 % CI) was 5.8 (4.9, 9.2) months for OaD versus 9.0 (5.5, 9.2) and 9.3 (9.0, 9.9) months for PRN and placebo, respectively. Showing a significant overall treatment effect (*p* = 0.038), the probability for EF-recovery was significantly higher for OaD versus placebo [hazard ratio (HR); 95 % CI 1.9; 1.2, 3.1; *p* = 0.011], but not for PRN versus placebo (*p* = 0.140). Of 57 OaD patients (41.0 %) with ED improved (by ≥1 IIEF-EF severity grade) at the end of DBT, 16 (28.1 % of 57) maintained this improvement through DFW and 27 (47.4 %) declined but maintained improvement from baseline after DFW.

**Conclusions:**

Data suggest that the use of tadalafil OaD can significantly shorten the time to EF-recovery post-nsRP compared with placebo.

**Electronic supplementary material:**

The online version of this article (doi:10.1007/s00345-014-1377-3) contains supplementary material, which is available to authorized users.

## Introduction

Erectile dysfunction (ED) can be a relatively common sequela after radical prostatectomy for localized prostate cancer [1–3], despite the use of nerve-sparing techniques (nsRP). Many men may not recover erectile function (EF) for more than 18 months post-nsRP [4, 5]. Rarely, time to EF-recovery can extend well beyond 2 years [6]. The rate of and time to EF-recovery post-nsRP can vary widely and may be influenced by a number of factors, including patient age, type of surgery, and treatment during the recovery phase [5–7].

Phosphodiesterase type 5 (PDE5)-inhibitors are generally well-tolerated and effective in the treatment of ED post-nsRP [1, 2, 8]. However, they are less effective in the post-nsRP population when compared to the general population, and the optimal time point for starting PDE5-inhibitor treatment is still undetermined [9].

To date, four randomized clinical trials (RCTs) have evaluated the impact of the early use of short-acting PDE5-inhibitors on EF-recovery in men post-nsRP. Sildenafil, vardenafil, and avanafil have all been shown to improve drug-assisted EF when given on demand (“pro-re-nata,” PRN) [10–12]. However, EF-recovery up to 1 year post-nsRP did not differ between sildenafil given once daily (OaD) or PRN [13].

For the long-acting PDE5-inhibitor tadalafil, an initial retrospective study in 92 patients showed that tadalafil OaD started early after robot-assisted laparoscopic nsRP was well tolerated and significantly improved EF compared to patients without PDE5-inhibitor treatment [14].

Montorsi et al. [15] have published a randomized, placebo-controlled trial evaluating the early use of tadalafil, given OaD or PRN, on both drug-assisted EF after 9 months of double-blind treatment (DBT) with tadalafil OaD or PRN and on unassisted EF (without PDE5-inhibitor support) after 6 weeks of drug-free washout (DFW) in men who developed ED post-nsRP. Tadalafil OaD improved drug-assisted EF-recovery, as measured by the proportion of patients achieving an International Index of Erectile Function-Erectile Function domain score (IIEF-EF) ≥22 at the end of DBT, while unassisted EF-recovery after DFW was not improved by tadalafil OaD or PRN [15].

This paper specifically addresses the effects of tadalafil OaD and PRN treatment on the time to recovery of EF (IIEF-EF ≥ 22) during the DBT period of this trial and the maintenance of treatment response after DFW.

## Materials and methods

### Patients

Adult men aged <68 years at the time of nsRP with normal preoperative EF who underwent nsRP for organ-confined, non-metastatic prostate cancer (Gleason score ≤7, prostate specific antigen <10 ng/mL) were enrolled between November 2009 and August 2011 in 50 centers from nine European countries and Canada (NCT01026818). Post-surgical inclusion criteria included the development of ED, as measured by a patient-reported Residual Erection Function (REF) score of ≤3 (“penis is hard enough for penetration but not completely hard”). This criterion was used because of the limited validity of IIEF-EF domain scores for direct EF assessment post-nsRP [15, 16]. Detailed eligibility criteria have been published [16].

### Trial design

This multicenter, Phase IV, randomized, double-blind, 3-arm, placebo-controlled parallel-group trial consisted of the following periods, as previously described (Supplementary Figure S1) [15]: screening (including nsRP surgery), 9-month randomized, double-blind, double-dummy treatment with 5 mg tadalafil OaD, 20 mg tadalafil PRN, or placebo (DBT); 6-week DFW; and 3-month open-label treatment with 5 mg/day tadalafil OaD (OLT, all patients).

### Outcome measures

#### IIEF-EF scores

The primary objective was to evaluate the efficacy of tadalafil OaD and tadalafil PRN, compared with placebo, in improving unassisted EF (EF after 6 weeks of DFW), as measured by the proportion of patients achieving an IIEF-EF score ≥22 at the end of DFW (primary outcome) [15]. An IIEF-EF ≥22 was required at screening (after cancer diagnosis, ≤6 weeks pre-nsRP). This cutoff was considered appropriate because many men with newly diagnosed prostate cancer claim to have unimpaired EF, but have IIEF-EF scores of 22–25 (mild ED) [15, 17]. *Time to EF*-*recovery* (additional secondary analysis pre-specified in the statistical analysis plan, finalized and approved before database lock) was defined as the time from baseline to reach an IIEF-EF ≥22 during DBT.

#### ED severity

IIEF-EF scores were categorized into the following ED severity categories: severe (0–10), moderate (11–16), mild (17–25), and normal (26–30) [18]. ED severity was assessed at baseline, end of DBT, and end of DFW. Improvement was defined as an IIEF-EF score of ≥1 category higher than baseline (or maintaining normal EF) at the end of DBT. Maintenance of treatment response, assessed for patients who improved ≥1 category after DBT, was defined as either maintaining this improved category until the end of DFW or declining after DBT but still maintaining a higher category at the end of DFW than at baseline.

### Statistical analysis

The planned sample size of 412 patients was based on the primary outcome (proportion of patients achieving IIEF-EF ≥22) [15]. All analyses were based on the intent-to-treat (ITT) population, including all randomized patients with baseline data and at least one post-baseline visit. Pre-specified treatment group comparisons were tadalafil OaD versus placebo and tadalafil PRN versus placebo.

The Kaplan–Meier product-limit method was used to estimate rates for the time to EF-recovery (IIEF-EF ≥ 22) including 95 % confidence intervals (CI). Only patients with IIEF-EF <22 at screening were included; patients not reaching IIEF-EF ≥22 were censored at the end of DBT. Hazard ratios (HRs) and *p* values were derived from a Cox proportional hazard model adjusting for treatment, age (<61 years vs. 61–68 years), and country.

IIEF-EF score changes from baseline were analyzed using a mixed model for repeated measures (MMRM) analysis, assuming an unstructured covariance structure and including visit, treatment, treatment-by-visit interaction, country, age group, and baseline as fixed effects, and patient and error as random effects. Adjusted least square means (LSmeans) and 95 % CIs were calculated from the model. A minimally clinically important difference (MCID), defined as ≥4 points difference in IIEF-EF [19], was used to determine the average needed treatment effect that has clinical relevance for patients.

For *p* values, a 5 % level of significance was used. Data were analyzed using the SAS 9.2 software (SAS Institute Inc., Cary, USA).

## Results

### Patient disposition and baseline characteristics

Of 583 patients screened, 423 were randomized: 139 (32.9 %) to tadalafil OaD, 143 (33.8 %) to tadalafil PRN, and 141 (33.3 %) to placebo (Supplementary Figure S2). Patients in the PRN group took a mean (SD) of 1.5 (0.95) tadalafil 20 mg tablets per week. Patient disposition, baseline demographics, and relevant disease characteristics were balanced in all 3 treatment groups (Table 1) [15]. As per inclusion criteria, all patients had to have IIEF-EF ≥22 pre-nsRP. Post-nsRP at baseline, 83.9 % of patients reported severe ED based on IIEF-EF scores (mean [standard deviation; SD] score 6.4 [5.81]) and >98 % reported an REF ≤3.

**Table 1:** Baseline characteristics and status post nsRP

Variable	Tadalafil OaD (*N* = 139)	Tadalafil PRN (*N* = 143)	Placebo (*N* = 141)
*Age* (*years*)			
Mean (SD)	58.6 (5.07)	57.5 (5.91)	57.6 (5.69)
<61 (*n*, %)	82 (59.0)	85 (59.4)	91 (64.5)
61–68 (*n*, %)	57 (41.0)	58 (40.6)	50 (35.5)
*Ethnicity* (*n*, %)			
Caucasian	137 (98.6)	141 (98.6)	138 (97.9)
*BMI* (kg/m^2^)			
Mean (SD)	26.6 (2.97)	26.9 (2.93)	27.1 (3.08)
*IIEF-EF*			
*N* with data	137	140	137
Mean (SD)	6.0 (5.80)	6.7 (5.57)	6.5 (6.08)
*ED severity* (*IIEF-EF categories*) (*n*, %)^a^			
Missing	2 (1.4)	2 (1.4)	4 (2.8)
Normal (26–30)	4 (2.9)	2 (1.4)	2 (1.4)
Mild (17–25)	5 (3.6)	8 (5.6)	9 (6.4)
Moderate (11–16)	9 (6.5)	10 (7.0)	11 (7.8)
Severe (0–10)	119 (85.6)	120 (84.5)	115 (81.6)
REF ≤ 3^b^	137 (98.6)	138 (97.2)	141 (100)
*nsRP approach* (*n*, %)			
Open surgery	68 (48.9)	65 (45.5)	56 (39.7)
Conventional laparoscopy	29 (20.9)	31 (21.7)	28 (19.9)
Robot-assisted laparoscopy	31 (22.3)	41 (28.7)	44 (31.2)
Other	11 (7.9)	6 (4.2)	13 (9.2)
*Total nerve-sparing score post-nsRP* (*n*, %)			
Perfect (2)	117 (84.2)	116 (81.1)	113 (80.1)
Not perfect (>2)	22 (15.8)	27 (18.9)	28 (19.9)

*BMI* body mass index, *ED* erectile dysfunction, *IIEF-EF* International Index of Erectile Function-Erectile Function, *N* total number of patients, *n* number of patients, *nsRP* bilateral nerve-sparing prostatectomy, *OaD* once a day, *PRN* “pro-re-nata”/on demand, *REF* residual erectile function, *SD* standard deviation

^a^Based on intent-to-treat population, excluding one patient from the tadalafil PRN group with no post-baseline data

^b^Two patients in the tadalafil OaD group and two patients in the tadalafil PRN group had missing values

### Time to EF-recovery during DBT

**Fig. 1:**
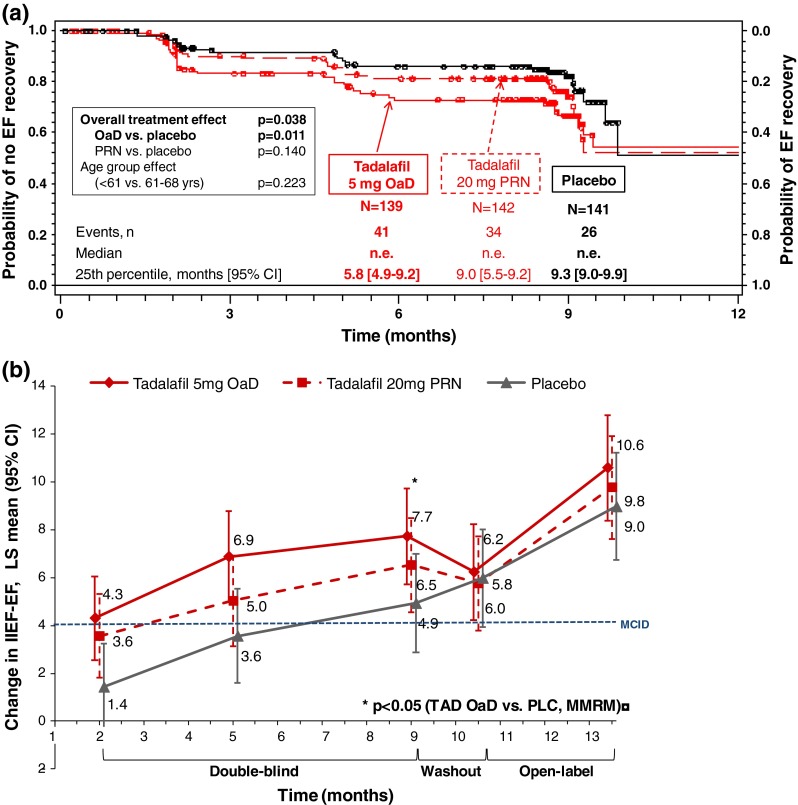
EF-recovery (IIEF-EF ≥ 22) and EF category improvement during DBT. *CI* confidence interval, *DBT* double-blind treatment, *EF* erectile function, *IIEF-EF* International Index of Erectile Function-Erectile Function domain, *LSmean* least square mean, *MCID* minimal clinically important difference, *MMRM* mixed model for repeated measures, *n* number of events, *N* number of patients, *n.e*. not estimable, *n.s.* not significant, *OaD* once a day, *PLC* placebo, *PRN* “pro-re-nata”/on demand, *TAD* tadalafil, *yrs* years. **a** Event (EF-recovery) was defined as change in IIEF-EF from <22 at screening to ≥22. *P* values are obtained from Cox proportional hazard model including terms for treatment, country, and age. **b** *p* value obtained from an MMRM model, assuming an unstructured covariance structure, including terms for visit, treatment, treatment-by-visit interaction, country, age group, and baseline IIEF-EF score as fixed effects, and patient and error as random effects. Previously published in: Montorsi et al. 2014 [15]

The proportion of patients achieving IIEF-EF ≥22 at any time point during DBT with OaD, PRN, and placebo was 29.5, 23.9, and 18.4 %, respectively. Based on the Kaplan–Meier analysis, 25 % of patients achieved EF-recovery (IIEF-EF ≥ 22) within 5.8 months for tadalafil OaD, 9.0 months for tadalafil PRN, and 9.3 months for placebo (Fig. 1a). Median time to EF-recovery could not be estimated as <50 % of patients achieved EF-recovery during the 9-month DBT period (Supplementary Figure S3). The Cox proportional hazard model showed a significant overall treatment effect (*p* = 0.038). Patients in the tadalafil OaD (but not PRN) group had a significantly higher probability for EF-recovery versus placebo (HR [95 % CI]: tadalafil OaD versus placebo: 1.90 [1.16, 3.12], *p* = 0.011; tadalafil PRN versus placebo: 1.47 [0.88, 2.47], *p* = 0.140). Age group had no significant effect on time to EF-recovery (*p* = 0.223; Supplementary Figure S4).

### LSmean IIEF-EF improvements

**Fig. 2:**
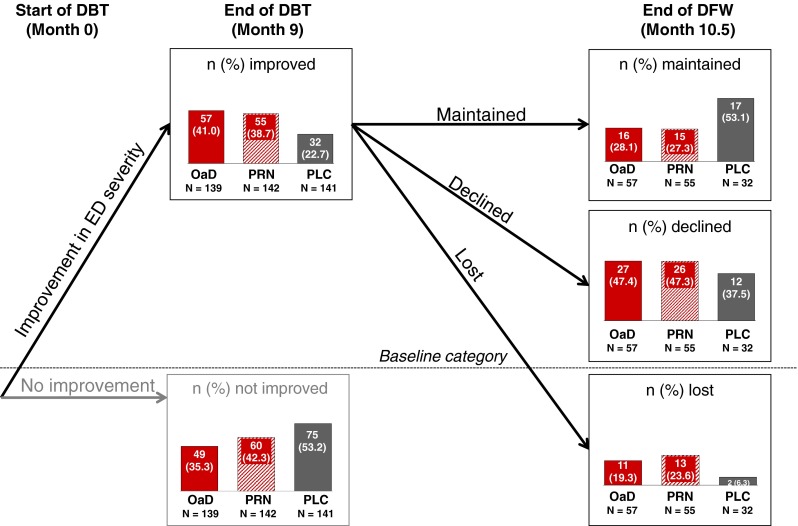
Improvement and maintenance of improvement from baseline in ED severity (based on IIEF-EF categories). *DBT* double-blind treatment, *DFW* drug-free washout, *ED* erectile dysfunction, *IIEF-EF* International Index of Erectile Function-Erectile function domain, *OaD* once a day, *PLC*, placebo, *PRN* “pro-re-nata”/on demand, *N* total number of patients, *n* number of patients. IIEF-EF scores defining ED severity categories: severe, 0–10; moderate, 11–16; mild, 17–25; normal, 26–30. *Improvement* was defined as reporting an IIEF-EF score of at least 1 category higher than baseline (or maintaining normal EF). *Improvement declined* was defined as reporting IIEF-EF scores at the end of DFW that were less than the end of the DBT but still at least 1 category higher than baseline. *Improvement maintained* was defined as reporting IIEF-EF scores at the end of DFW that were at least as high or higher than scores at the end of DBT. *Improvement lost* was defined as IIEF-EF scores that were less than or equivalent to ED severity at baseline. All percentage are relative to the size of each treatment group (“N,” provided below *each bar*) not relative to the overall population. Missing data: For 33 patients (23.7 %) in the tadalafil OaD group, for 27 (19.0 %) in the tadalafil PRN group, and for 34 (24.1 %) in the placebo group, improvement could not be calculated because the patient either discontinued during DBT or had missing IIEF-EF scores at baseline and/or month 9. For three patients (5.3 %) in the tadalafil OaD group, one (1.8 %) in the tadalafil PRN group, and one (3.1 %) in the placebo group, maintenance of improvement could not be calculated because the patients either discontinued during DFW or had missing IIEF-EF scores at month 10.5

LSmean IIEF-EF improvement during DBT significantly exceeded the MCID (Lower 95 % CI LSmean ΔIIEF-EF ≥ 4) at month 5 in the tadalafil OaD treatment group (LSmean [95 % CI]: 6.9 [5.0, 8.8]) and month 9 in the tadalafil PRN treatment group (6.5 [4.6, 8.5]) (Fig. 2). For placebo, LSmean IIEF-EF did not significantly exceed the MCID before month 10.5 (end of DFW: 6.0 [3.9, 8.0]). The treatment effect versus placebo was statistically significant for tadalafil OaD only (LSmean difference [95 % CI]: 2.8 [0.8, 4.8]; *p* = 0.007) at month 9.

### ED severity improvement and maintenance of improvement

At baseline (randomization), 83.9 % of patients overall had severe ED (IIEF-EF 0–10; Table 1). During DBT, improvement in ED severity by ≥1 severity grade was achieved by 41.0 % of all tadalafil OaD patients, 38.7 % of all tadalafil PRN patients, and 22.7 % of patients on placebo (Fig. 2). In all groups, the majority of those patients who had improved during DBT maintained an improvement of ≥1 severity grade from baseline through DFW (improvement maintained, Fig. 2). For tadalafil OaD (Fig. 2), 43 of 57 improved patients (75.4 %) were still improved from baseline after DFW, including 16 patients (28.1 %) who maintained the improvement they had reached at the end of DBT and 27 patients (47.4 %) who declined but still maintained improvement from baseline after DFW.

## Discussion

This trial was the first RCT in patients with established ED post-nsRP which investigated the effect of early treatment with tadalafil OaD and PRN on EF-recovery. As previously reported, tadalafil OaD significantly improved drug-assisted EF-recovery, as measured by the proportion of patients achieving IIEF-EF ≥22 at the end of DBT (25.2 % of OaD patients versus 14.2 % in placebo group; *p* = 0.016; Supplementary Figure S3). Unassisted EF-recovery after DFW was not improved by tadalafil OaD or PRN [15]. Here, we show that tadalafil OaD (but not PRN) significantly shortened the time to EF-recovery during DBT when compared with placebo: with placebo, it took 9.3 months until 25 % of patients had reached EF-recovery; this period was shortened by 3.5 months (i.e., to 5.8 months) in the tadalafil OaD treatment group. An early start of penile rehabilitation does seem to be important; Mulhall et al. [20] showed that patients who started PDE5-inhibitor treatment early post-nsRP reached significantly higher mean IIEF-EF scores than patients who started later at ≥6 months post-nsRP (*p* < 0.0001).

EF-recovery rates during 9-month DBT were <50 % in all treatment groups, which is in line with the published data on natural EF-recovery post-nsRP. Without treatment, time to EF-recovery averages 18 months [4] and can extend well beyond 2 years [5, 6]. In a study by Gallina et al. [21], only 35.8 % of untreated patients reached EF-recovery (IIEF-EF ≥ 22) after an average of 26.8 months post-nsRP.

However, LSmean IIEF-EF improvement in the tadalafil OaD group significantly exceeded the MCID (ΔIIEF-EF ≥ 4 [19]) already at month 5 of DBT, as compared to month 9 for tadalafil PRN; improvement with placebo did not significantly exceed the MCID before month 10.5 (end of DFW). At the end of DBT, the treatment effect versus placebo was statistically significant for tadalafil OaD only (*p* = 0.007).

Chronic (daily) dosing of tadalafil, but not PRN treatment, will lead to steady state PDE5-inhibition [22] which may be associated with prolonged (continuous) periods of increased tissue oxygenation during the post-operative regenerative process. Preclinical data suggest that chronic low-dose administration may protect from structural changes of penile cavernous corpora and is associated with EF enhancement [23–25].

None of the other RCTs on PDE5-inhibitors post-nsRP has reported time to EF-recovery data based on Kaplan–Meier analysis. However, data from 2 non-RCTs indicated that PDE5-inhibitor treatment may shorten time to EF-recovery [26, 27]. Bannowsky et al. [26] reported a significant difference in time to EF-recovery between patients receiving nightly low-dose sildenafil for up to 12 months when compared with patients receiving no treatment (*p* < 0.001). In agreement with these results, Briganti et al. showed that patients receiving any PDE5-inhibitor (OaD or PRN) achieved significantly higher 3-year EF-recovery rates (IIEF-EF ≥ 22) than patients receiving placebo (72 vs. 38 %, *p* ≤ 0.001, Kaplan–Meier analysis). For the overall population studied, no significant difference was observed between OaD and PRN treatment. However, patients with an intermediate risk of ED (66–69 years or IIEF-EF 11–25, and Charlson Comorbidity Index ≤1), who shared key criteria with our patient population of low/intermediate ED risk (average patient ≤61 years; IIEF-EF ≥ 22 at baseline), achieved significantly higher 3-year EF-recovery rates with OaD compared to PRN treatment (74 vs. 52 %; *p* = 0.02) [27]. As suggested by Castiglione et al. [28], the effect of PDE5-inhibitor treatment post-nsRP may be maximal in patients with intermediate ED risk. To date, trials have focused on populations with low ED risk [11, 13, 15].

In our trial, age group had no significant effect on the time to EF-recovery during DBT or on the proportion of patients achieving EF-recovery during DBT [15]. However, after DFW (Month 10.5), younger patients (<61 years) were significantly more probable to achieve EF-recovery than older patients (*p* = 0.020; [15]). These results are in line with literature.

A 2010 study by Briganti et al. [29] showed that younger patients (≤65 years) were more likely to recover EF (IIEF-EF ≥ 22) than older patients. A meta-analysis by Kilminster et al. and studies by Nelson et al. and Gallina et al. also showed that younger patients were significantly more likely to recover EF post-nsRP than older patients [5, 7, 21].

A clear limitation of the current trial was that the 9-month DBT phase was too short for full assessment of EF-recovery. We cannot exclude that the treatment effect of tadalafil OaD may be lost by the end of 2 years due to spontaneous EF-recovery in the placebo arm. Valid statistical analysis of time to EF-recovery could not be performed on IIEF-EF data collected after DBT (i.e., after DFW at month 10.5 or OLT at month 13.5) due to the break in ED treatment for 6 weeks. The significant treatment effect on EF-recovery was lost during the DFW. However, after 3-month OLT with tadalafil OaD (month 13.5), the proportion of patients with EF-recovery increased in all treatment groups (32.4 % in the tadalafil OaD group; 33.1 % in the tadalafil PRN group; and 27.0 % in the placebo group) [15]. Further, the results of the Briganti study indicate that the significant effect of OaD treatment on time to recovery may persist after longer follow-up periods (up to 3 years) [27]. In this context, the demonstrated maintenance of tadalafil’s treatment effect could play an important role in future studies that allow for longer treatment or follow-up periods. Even after DFW, over 75 % of patients treated with tadalafil OaD maintained an improvement in ED severity from baseline.

In conclusion, patients taking tadalafil OaD (but not those taking PRN) significantly shortened the time to EF-recovery during DBT when compared with placebo. No statistically significant difference in time to EF-recovery was observed between younger and older patients. These data suggest that tadalafil OaD, if started early, may accelerate EF-recovery post-nsRP.

## Electronic supplementary material

Below is the link to the electronic supplementary material.

Supplementary material 1 (PDF 333 kb)

## Abbreviations

CI: Confidence interval
DBT: Double-blind treatment
DFW: Drug-free washout
ED: Erectile dysfunction
EF: Erectile function
HR: Hazard ratio
IIEF-EF: International Index of Erectile Function-Erectile Function domain score
ITT: Intent-to-treat
LSmeans: Least square means
MCID: Minimally clinically important difference
MMRM: Mixed model for repeated measures
nsRP: Nerve-sparing radical prostatectomy
OaD: Once daily
OLT: Open-label OaD treatment
PDE5: Phosphodiesterase type 5
PRN: “Pro-re-nata,” on demand
RCTs: Randomized clinical trails
REF: Residual erection function
SD: Standard deviation
