# Supplementary material for: Effects of tadalafil once daily or on demand versus placebo on time to recovery of erectile function in patients after bilateral nerve-sparing radical prostatectomy
Source: World J Urol. 2014 Aug 26;33(7):1031–8. doi: 10.1007/s00345-014-1377-3 (PMC4480825; doi:10.1007/s00345-014-1377-3)
Supplement: Supplementary file 1 — Supplementary material 1 (PDF 333 kb) [file 345_2014_1377_MOESM1_ESM.pdf]

Electronic Supplementary Material:

World Journal of Urology

Effects of Tadalafil Once Daily or On-Demand versus Placebo on Time to Recovery of Erectile Function in Patients after Bilateral Nerve-Sparing Radical Prostatectomy

Authors: Ignacio Moncada, Fermín R. de Bethencourt, Enrique Lledó-García, Javier Romero-Otero, Carmen Turbi, Hartwig Büttner, Carsten Henneges, Juan I. Martinez Salamanca

Corresponding author: Ignacio Moncada, Department of Urology, Hospital La Zarzuela Madrid, Spain. E-mail: ignacio@moncada.name

Supplementary Figure S1: Trial design

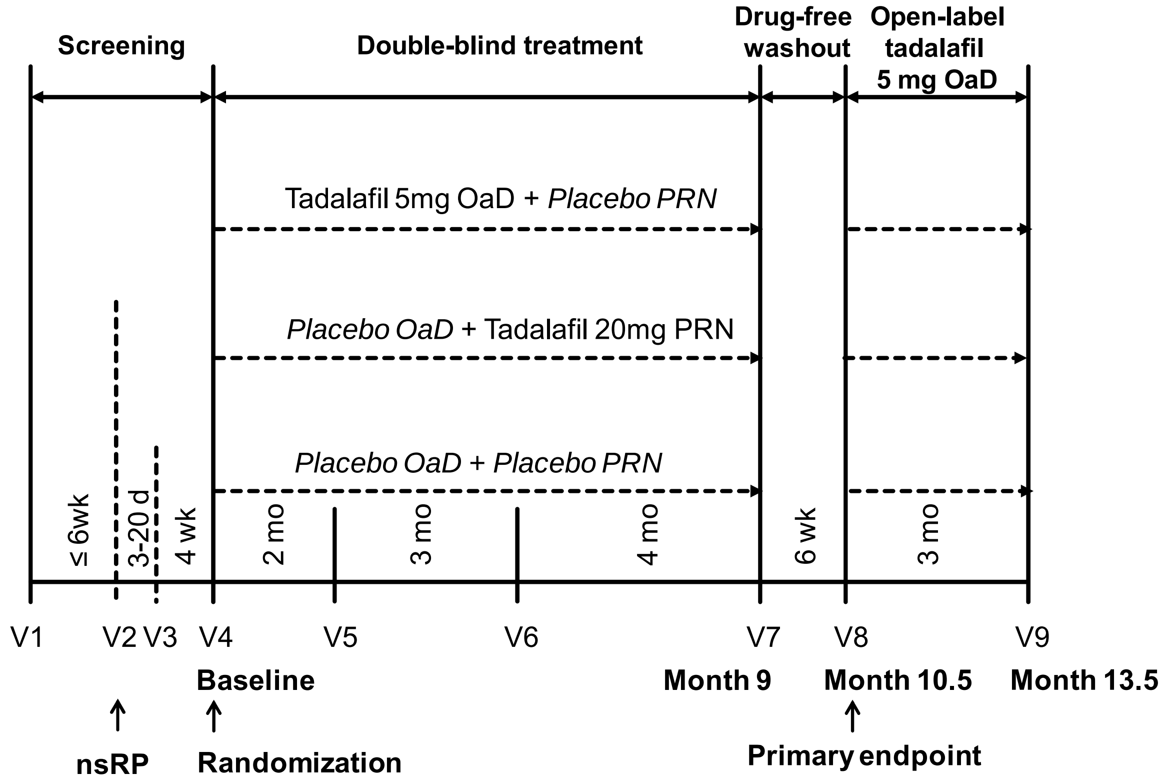

Abbreviations: nsRP, bilateral nerve-sparing radical prostatectomy; d, day; mo, month; OaD, once a day; PRN, “pro-re-nata”/on-demand; V, visit; wk, week.

Previously published in: Montorsi et al. 2014 [15].

Supplementary Figure S2 Patient Disposition

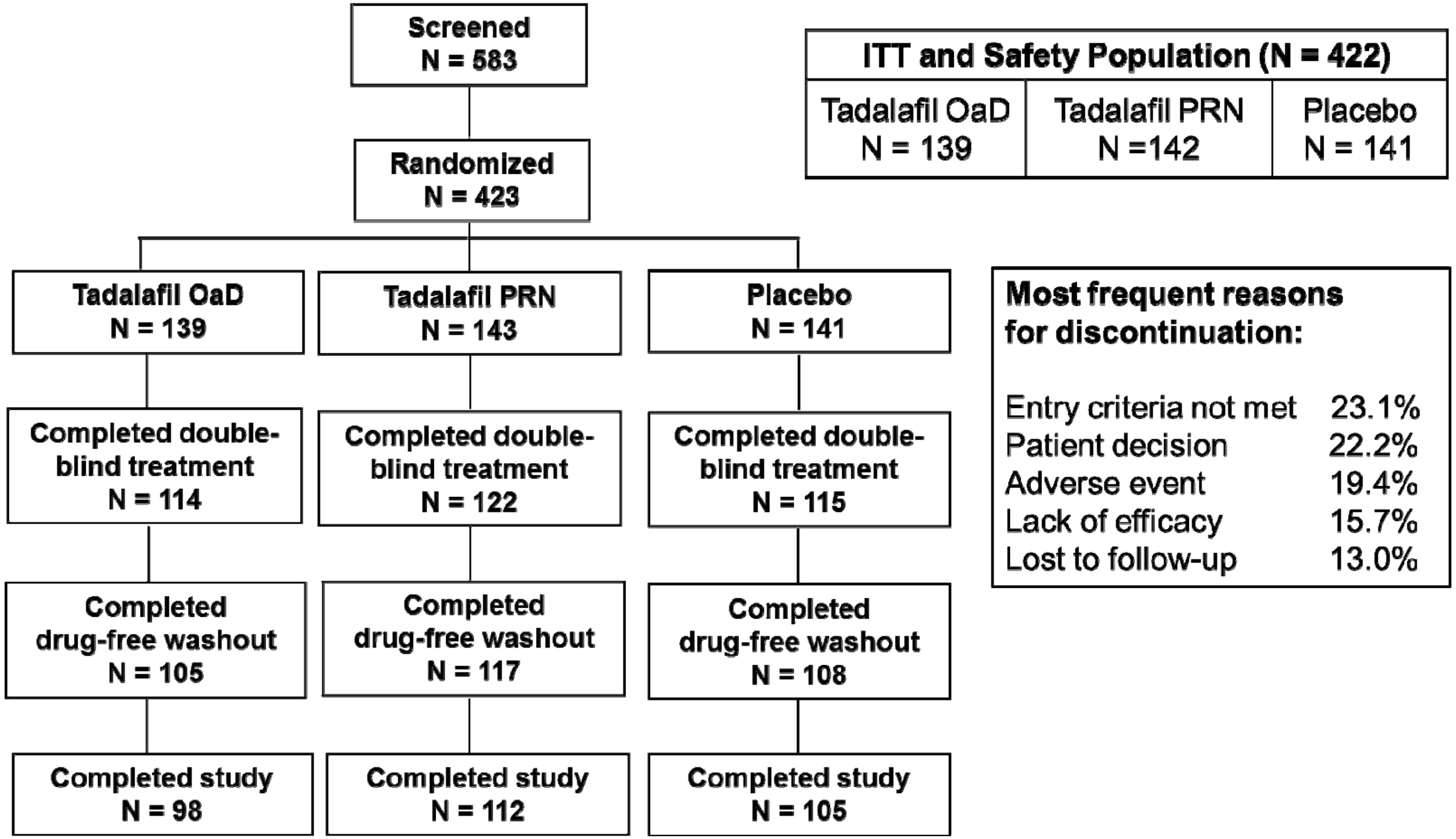

Abbreviations: ITT, intention-to-treat; OaD, once a day; PRN, “pro-re-nata”/on-demand; N, number of patients.

Previously published in: Montorsi et al. 2014 [15].

Supplementary Figure S3 Percent Patients Achieving IIEF-EF Score ≥22

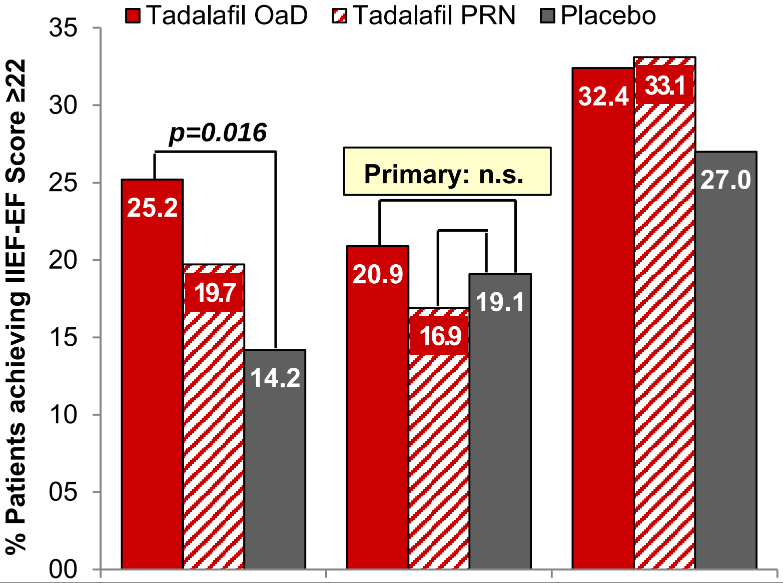

	Month 9	Month 10.5	Month 13.5
	Odds Ratio [95% CI]; *p-value*		
OaD vs. Placebo	**2.15 [1.16, 3.99]; *0.016***	1.14 [0.63, 2.06]; *0.675*	1.34 [0.79, 2.28]; *0.273*
PRN vs. Placebo	1.50 [0.79, 2.85]; *0.210*	0.89 [0.48, 1.65]; *0.704*	1.35 [0.80, 2.29]; *0.259*

Abbreviations: CI, confidence interval; IIEF-EF, International Index of Erectile Function-Erectile Function domain score; n.s., not significant; OaD, once a day; PRN, “pro-re-nata”/on-demand.

Previously published in: Montorsi et al. 2014 [15].

Supplementary Figure S4 Cox Proportional Hazard Model on Time to EF-Recovery (IIEF-EF≥22) during DBT

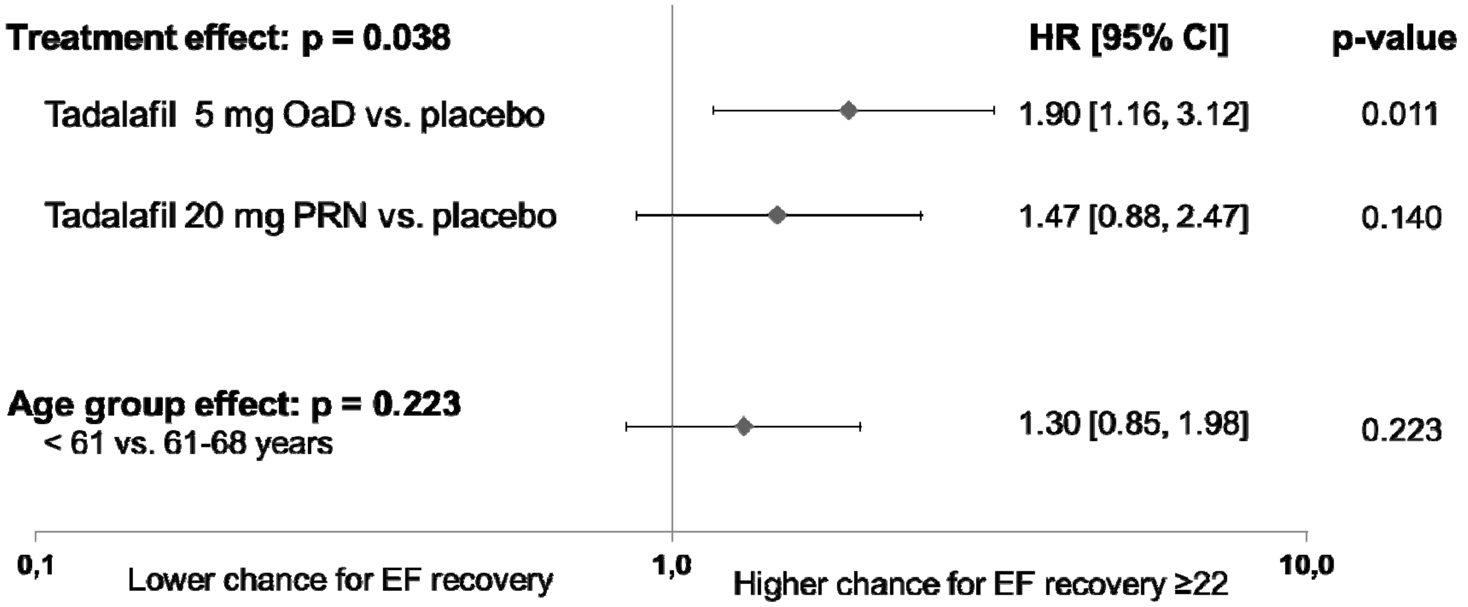

Abbreviations: CI, confidence interval; DBT, double-blind treatment; EF, erectile function; HR, hazard ratio; IIEF-EF, International Index of Erectile Function-Erectile Function domain score; OaD, once a day; PRN, “pro-re-nata”/on-demand.
